# Analysis of in-person general practice respiratory consultations: assessing translatability to telehealth

**DOI:** 10.3399/BJGPO.2023.0073

**Published:** 2023-10-04

**Authors:** Sunayana Raghuraman, Jayashanthi Ramarao, Jared Lane, Kanesha Ward, Annie Lau

**Affiliations:** 1 Centre for Health Informatics, Australian Institute of Health Innovation, Macquarie University, Sydney, Australia; 2 Westmead Hospital, Western Sydney Local Health District, NSW Health, Sydney, Australia; 3 Liverpool Hospital, South Western Sydney Local Health District, NSW Health, Sydney, Australia

**Keywords:** COVID-19, digital, general practice, referral and consultation, remote, respiratory, telemedicine

## Abstract

**Background:**

The COVID-19 pandemic saw many GPs adopt telehealth as a consultation modality to minimise disease transmission. Patients presenting with respiratory ailments were particularly affected by this transition, given the overlap of general respiratory symptoms with those of COVID-19. It is unclear if the rapid transition to telehealth has compromised the ability to conduct certain tasks that were possible during in-person consultations.

**Aim:**

To investigate the extent to which tasks observed during in-person GP consultations are replicable in telehealth, focusing on patients with respiratory concerns.

**Design & setting:**

Twenty-six respiratory consultations were extracted from a database of 281 consultations collated from various general practices in the UK.

**Method:**

Interactions between GPs and respiratory patients were assessed through in-depth transcript review and de-identified video analysis. Then, tasks performed and physical artefacts used during the consultations were identified and ranked in terms of their translatability to telehealth, using a newly developed scoring system.

**Results:**

Overall, the translatability to telehealth score for these respiratory consultations was 6.7/10, suggesting that many tasks can be replicated over telehealth, but that they might require additional physical artefacts to support this. However, some tasks are not currently amenable to telehealth (for example, auscultation).

**Conclusion:**

While many aspects of respiratory consultations are replicable over telehealth, some tasks cannot be replicated at this stage.

## How this fits in

Given the increasing role of telehealth as a healthcare modality, it is insightful to identify the tasks that are present during in-person consultations between GPs and patients, and to assess how these can be replicated in telehealth. To the authors’ knowledge, there is no current research that investigates how translatable to telehealth different in-person respiratory consultation tasks are. This study aims to address this gap by not only evaluating the translatability of various clinical tasks performed for respiratory patients in general practice, but also to assess whether a scoring system can be developed to quantify this translatability.

## Introduction

Telehealth is a consultation modality that entails clinical interactions occurring remotely through either video or audio calls. While telehealth was used by healthcare practitioners before COVID-19, the pandemic resulted in a rapid uptake of telehealth across the globe within a short period of time.^1–4^ Given the overlap in symptoms with many respiratory conditions and those of COVID-19, numerous patients with respiratory ailments had to resort to telehealth consultations to minimise disease transmission. In the UK, nearly one in five patients are diagnosed with a respiratory condition in their lifetime.^5^ One cohort study of patients in South West England showed that since the COVID-19 pandemic, the number of patients presenting to their GP with a respiratory complaint had increased by 229%, with a 105% increase in home visits, 92% increase in office visits, 250% increase in phone consultations, and a 1574% increase in video/email (that is, telehealth) consultations.^6^

Respiratory conditions (for example, viral illnesses, asthma, and chronic obstructive pulmonary disease [COPD]) are among the most common reasons for a patient to visit a general practice.^7–9^ Some respiratory ailments can be debilitating, impede quality of life, impact mental health, and, if improperly managed, increase the risk of morbidity and mortality, subsequently burdening the healthcare system.^9,10^ As gatekeepers to the healthcare system, GPs are well situated to monitor and manage respiratory conditions to prevent exacerbations and reduce hospital admissions.^11–13^

A new body of literature investigating the efficacy of telehealth for patients with respiratory concerns during the COVID-19 pandemic is slowly emerging. Studies by McGee *et al* and Phillips *et al*, to name two, have found that telehealth is an effective modality for delivering health care.^14,15^ However, to the authors’ knowledge, no studies have investigated the tasks performed during in-person respiratory consultations in general practice, or analysed which aspects are translatable to telehealth and what physical objects/artefacts are required to support this.

The aim of this study was to provide an insight into the tasks and physical artefacts used during in-person GP consultations, and subsequently, to determine if these are translatable to the context of telehealth in respiratory patients. This study is unique in that it evaluates actual interactions between GPs and patients, rather than relying on self-reported data, which can be amenable to recall or confirmation bias.^16^

## Method

### Study design

This study is a secondary analysis of both written transcripts and de-identified videos of primary care consultations between GPs and patients in the UK. The data were collected in a project ethically approved by the NHS entitled *‘Harnessing resources from the internet to maximise outcomes from GP consultations (HaRI) study: a mixed methods study’*.^17^

### Data collection

The original dataset of 281 consultations (in transcripts and videos) were retrieved from 10 GPs across eight general practices in different locations across South East England in 2017. Consultations between GPs and patients were de-identified, transcribed verbatim, and summarised into an IBM SPSS Statistics metadata file. Two researchers reviewed the transcripts and employed inclusion and exclusion criteria to isolate relevant respiratory consultations (see Supplementary Figures S1 and S2). The inclusion criteria comprised consultations:

- obtained from the original HaRI database;
- that discussed a respiratory concern; and
- where consent was obtained for the use of both written transcripts and de-identified videos.

The exclusion criteria comprised consultations where:

- consent was declined; or
- no clear discussion surrounding a respiratory complaint occurred.

### Data analysis

#### Patient privacy and confidentiality

To maintain privacy and confidentiality, a custom-made software program was developed and applied to the consultation videos to obscure patients’ and clinicians’ faces.

#### Descriptive analysis

Descriptive statistical analysis detailing patient demographics was compiled from the isolated respiratory transcripts (see Supplementary Table S1).

#### Video and transcript analysis

Two independent reviewers conducted an in-depth analysis of the written transcripts using a similar framework to that of Kocabelli *et al*.^18^ This study assessed various patient transcripts and transcribed every single event that occurred.^18^ Similarly, inductive analysis was employed in this study by identifying and documenting every task that occurred and what physical artefacts were used for these tasks. The identified tasks were documented and coded (that is, grouped) using NVivo (version 12) software . Furthermore, corresponding de-identified videos of the transcripts were analysed to note any additional tasks and/or artefacts that were not identified during the transcript review.

The identified physical artefacts were subcategorised into objects that were likely to be accessible in a patient’s home (and thus amenable to use in telehealth consultations) and objects that were not. This was based on the availability of the equipment in an average pharmacy in a developed nation (for example, Boots in the UK).

#### Translatability to telehealth analysis

From the inductive analysis conducted above, it was noted that two basic domains underpinned whether the observed tasks would be amenable to telehealth: whether clinical expertise was required (for example, physical examination skills) and whether supplementary physical artefacts were required (for example, thermometer). A separate co-study by one co-author found that these domains underpinned the analysis of cardiovascular transcripts as well.^19^

Based on this, a scoring system was developed by four researchers to investigate the extent to which various tasks were amenable to telehealth. This scoring system was inspired by Croymans *et al*,^20^ who assessed various presenting complaints and categorised them into health conditions that were suitable for telehealth, compared with those that were not. This prompted the authors of the present study to contemplate whether various clinical tasks, rather than conditions, could be categorised, to determine if certain tasks were more amenable to telehealth than others.

This scoring system was developed through collaborative discussions between the researchers, a review of relevant literature, and in-depth analysis of the transcripts. The following steps were used to develop the scoring system, which ultimately provided a ‘translatability to healthcare score’:

1. Each task was rated based on the requirement of ‘clinical endorsement’. A score of 1 was assigned when medical accreditation/training was required (for example, prescribing), and a score of 5 was assigned where clinical endorsement was not necessarily required (for example, formal greeting) (Table 1).
2. Each task was then rated based on the need for physical artefacts/interactions for execution. A score of 1 was assigned for physical artefacts currently unlikely to be replicated over telehealth (for example, auscultation), and a score of 5 was assigned where physical artefacts were not required and thus readily amenable to telehealth (for example, history taking).
3. The scores from steps 1 and 2 were combined to provide a translatability to telehealth score (x/10) (Table 2). A higher score equated increased translatability to telehealth.
4. Based on the translatability to telehealth score, each task was categorised into a virtual care solution (refer to Table 3).

**Table 1.** Detailed metrics used to score translatability of in-person tasks to telehealth

Metric 1: Clinical endorsement score	
**Score**	**Description**
1/5	Medical expertise is necessary for tasks that can only be performed in person (for example, giving flu injections and auscultation^a^)
2/5	In-person medical expertise is preferred, but some digital solutions are available in an outpatient setting, although not in the patient’s home (for example, outpatient spirometry)
3/5	Medical endorsement is required for interpretation of results that the patient can collect in their homes (for example, home temperature monitoring), as well as for tasks that have current digital solutions (for example, provide electronic referrals, electronic prescriptions to pharmacy, and medical certificates)
4/5	Medical expertise is required for tasks, such as targeted history taking, but no specific equipment is required, making it easy to perform via telehealth
5/5	Medical expertise is not necessarily required to complete this task (for example, formal greeting)
**Metric 2: Physical artefacts or physical interactions score**	
**Score**	**Description**
1/5	Requires physical artefacts for execution in a manner that is currently not easily translatable via telehealth (for example, auscultation^a^)
2/5	Requires equipment that is currently not accessible in the home, but results could be discussed over telehealth (for example, X-ray and spirometry)
3/5	Requires equipment that is easily purchasable in most pharmacies, as verified against pharmacy catalogues in the UK/Australia; that is, Boots and Chemist Warehouse (for example, purchasing a peak flow metre)
4/5	Requires equipment that is relatively easily accessible in many homes, and thus is able to be translated via telehealth (for example, thermometer)
5/5	Does not require any equipment, and thus is readily translatable over telehealth (for example, discussing smoking status)

a In some circumstances, auscultation may be considered a 2/5 as new technology for remote auscultation is developing. However, this technology is expensive and not yet readily available; thus, it has been rated 1/5 in this circumstance.

**Table 2.** Translatability to telehealth score interpretation

Metric 1: Clinical endorsement score	Metric 2: Physical artefacts or interactions score				
	5	4	3	2	1
5	10	9	8	7	6
4	9	8	7	6	5
3	8	7	6	5	4
2	7	6	5	4	3
1	6	5	4	3	2

9–10/10 [RED] Easily translatable over telehealth with almost no additional equipment being required = Type 5.

7–8/10 [ORANGE] Relatively easy to translate over telehealth, with minimal but easily accessible equipment required = Type 4.

5–6/10 [YELLOW] can be translated over telehealth but may require the patient to acquire their own additional equipment to do so = Type 3.

4/10 [GREEN] Can be translated over telehealth but may require the patient to undergo additional steps e.g.(for example, outpatient investigations) = Type 2.

2–3/10 [PINK] Not amenable to being replicated over telehealth at this stage = Type 1.

**Table 3.** Virtual care solution types

Virtual care solution type	Interpretation
Type 5	Tasks that are easily translatable over telehealth (for example, history taking and test result interpretation)
Type 4	Tasks that are relatively easy to translate to telehealth with minimal effort and easily accessible equipment (for example, patients recording own temperature at home and communicating findings to GP)
Type 3	Tasks that are moderately translatable to the context of telehealth but may require the patient to purchase additional physical artefacts available in a pharmacy to support this (for example, purchasing a peak flow metre)
Type 2	Tasks that cannot necessarily be supported with physical artefacts at home, but investigations can be performed as an outpatient and discussed virtually (for example, chest X-ray interpretation and spirometry results)
Type 1	Tasks that require in-person consultations to effectively execute, and thus are not amenable to telehealth at this stage (for example, auscultation)

Thus, the scoring system was developed after evaluating the data (inductive analysis), and was then re-applied to the clinical tasks to see if they could be categorised using this method (deductive analysis). See Supplementary Figures S1 and S2 for diagrammatic representation of methods.

## Results

### Patient and consultation characteristics

After applying inclusion and exclusion criteria to the dataset of 281 transcripts, 26 transcripts discussing respiratory illnesses in a general practice setting were extracted. These transcripts included acute and chronic presentations across all age ranges, with a variety of objective and subjective measures of respiratory function (see Supplementary Table S1). Please refer to Supplementary Table S2 for examples of raw data collection methodology.

### Physical artefacts used and their replicability in patients’ homes

Sixteen physical artefacts were identified, of which eight (50%) were deemed amenable to being replicated in telehealth, while the other eight (50%) were not, owing to equipment availability or requirement for operator expertise (see Supplementary Tables S3–S5). Please refer to Supplementary Table S6 for raw data collection.

**Figure 2.**
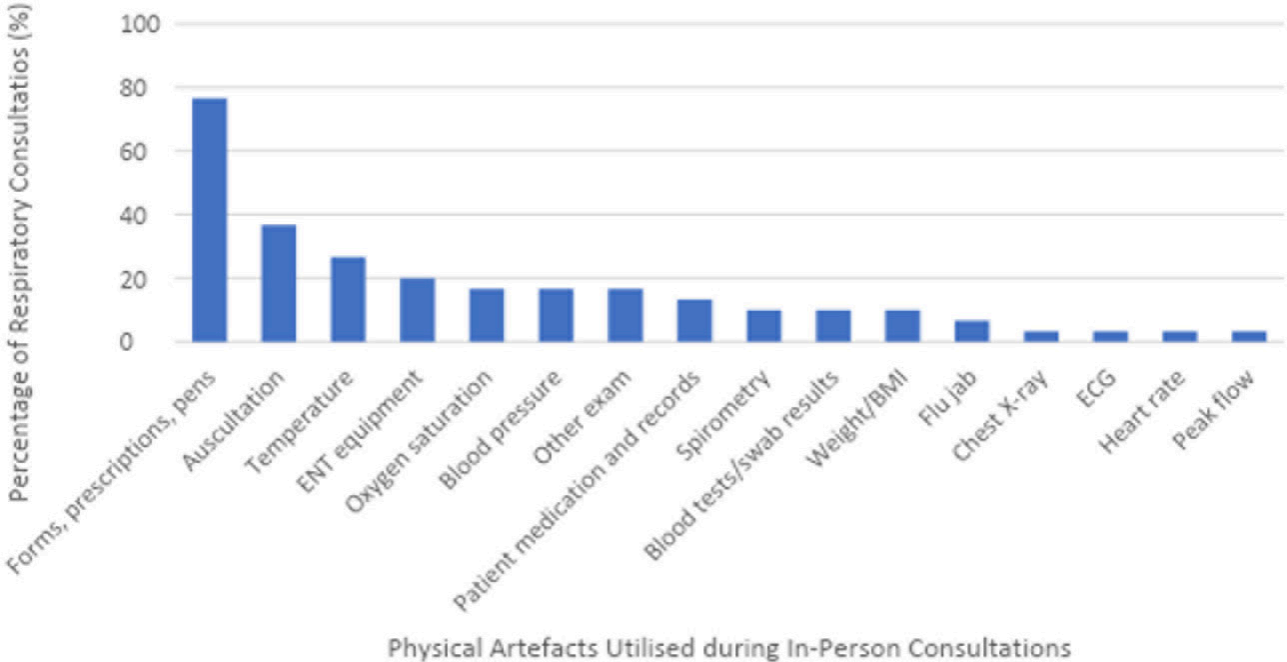
Figure 2Frequency of physical artefacts used in respiratory consultations (*n* = 26). Some consultations incorporate multiple artefacts within the same encounter. BMI = body mass index. ECG = electrocardiogram. ENT = ear, nose, and throat.

### Tasks performed and translatability to telehealth

Supplementary Table S7 outlines the tasks identified during in-person consultations relating to respiratory concerns. See Supplementary Table S3 for examples of rationales regarding scoring for various tasks.

Across these 20 tasks, the mean score for the telehealth metrics were:

- Requiring healthcare endorsement = 3.1/5 (where 1 = clinical expertise is necessary; and 5 = clinical expertise is not required).
- Requiring physical artefacts/interactions = 3.6/5 (where 1 = requires physical artefacts/interactions that are not translatable to telehealth; and 5 = no physical artefacts/interactions required).
- Translatability to telehealth score = 6.7/10 (where 1 = not replicable to telehealth at this stage; and 10 = easily replicable over telehealth at this stage).

Each of these 20 tasks were then categorised into a virtual care solution type (see Table 3). Overall, the proportions of virtual care solution types were:

- Type 1 (tasks not translatable to telehealth, thus requiring in-person consults) = 15% (*n* = 3/20); that is, auscultation; ear, nose, and throat (ENT) examination; and flu injections.
- Type 2 (tasks that cannot be performed at home, but can be performed as an outpatient with results discussed virtually) = 10% (*n* = 2/20); that is, spirometry results; blood tests; and X-rays.
- Type 3 (moderately translatable to telehealth but may require supplementation with further physical artefacts) = 10% (*n* = 2/20); that is, completing power of attorney paperwork and measuring peak flow.
- Type 4 (easily translatable over telehealth with easily accessible equipment) = 30% (*n* = 5/20); that is, measuring vital signs; providing medical certificates; and prescribing medications.
- Type 5 (easily translatable over telehealth without the need for physical artefacts) = 40% (*n* = 8/20); that is, formal greetings/farewells; history taking; patient education; and safety netting.

Please refer to Supplementary Table S8 for a summary of which consultation IDs involved various clinical tasks.

### Clinical tasks performed during in-person consultations

**Figure 1.**
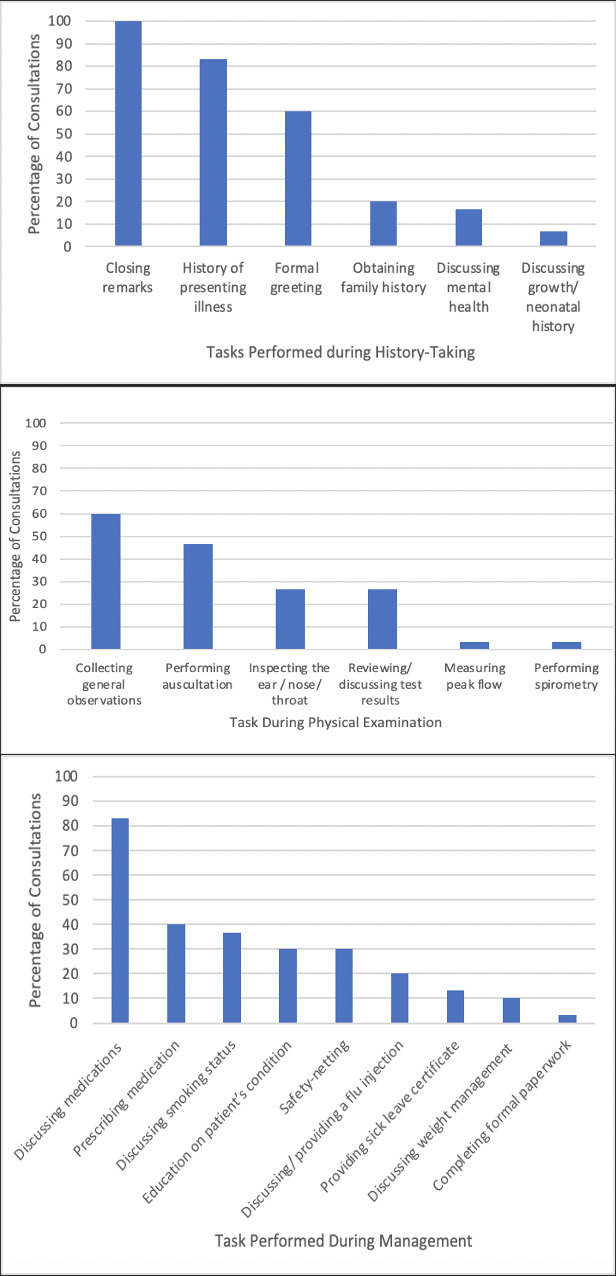
Frequency of clinical tasks performed in respiratory consultations (*n* = 26). Some consultations incorporate multiple tasks within the same encounter.

Figure 1 summarises the frequency of the tasks described in Supplementary Table S7 in history taking, examination, and management, respectively.

## Discussion

### Summary

The mean translatability to telehealth score was 6.7/10, suggesting many tasks can be replicated over telehealth, but may require additional physical artefacts. It was noted that 50% (*n* = 8/16) of the physical artefacts used were deemed to be accessible in patients’ homes or available for purchase at most pharmacies, while the other 50% (*n* = 8/16) were not. The physical artefacts that were deemed possible to replicate in a patient’s home included: pens/paperwork/prescriptions (for example, online scripts), thermometer, oxygen saturation monitor, blood pressure cuff, patient medication lists/patient-made notes, weighing scale, heart rate monitor, and peak flow metre. Physical artefacts/tasks that were deemed to be difficult to replicate in a patient’s home included auscultation (as it requires clinical expertise), ENT examination (for example, otoscopy), spirometry, blood tests, flu injections, chest X-rays, and electrocardiograms (ECGs). These findings could assist GPs conducting remote respiratory consultations, by helping them consider what additional tasks/artefacts a patient requires to achieve a successful respiratory telehealth consult. Please refer to Supplementary Table S9 for a breakdown of consultation durations and the number of physical interactions occurring during each consultation.

### Strengths and limitations

This study adopted a unique methodology that comprised of analysing actual GP–patient interactions during in-person consultations, rather than questionnaires or interviews, to facilitate objective analysis of tasks and reduce the risk of recall bias. Furthermore, the analysed consultations involved variable sociodemographic features and both acute and chronic respiratory complaints, which assists with extrapolation of the findings to a wider population. Finally, the scoring system developed is unique and, to the authors’ knowledge, the first to quantify the translatability of tasks to telehealth.

While the consultations incorporated patients with various sociodemographic characteristics, they were all located in South East England. This impacts the ability to extrapolate the findings to UK primary care overall, as different areas of the UK have variable prevalence and mortality rates of different respiratory conditions. For example, pneumonia had a higher number of deaths in South East England compared with lung cancer and COPD; whereas COPD and lung cancer were associated with a higher number of deaths than pneumonia in North West England.^21^

Some aspects of the consultations were performed behind curtains for patient privacy, and thus were unable to be adequately evaluated. Furthermore, the categorisation of some tasks can be subjective. For example, flu vaccinations were given a translatability to telehealth score of 2/10, as the observed consultation video involved in-person interactions to administer the injection. However, patients could be referred to a pharmacy and still obtain the same treatment (that is, a translatability to telehealth score of 3/10). Additionally, some of the task categorisations assume that the consumer has access and financial means to purchase additional physical artefacts if needed. This is a limitation, as some regions that would benefit from telehealth (for example, rural/regional communities) may be unable to easily access resources required to supplement virtual appointments. The accuracy of measurements taken by patients in their homes can also be difficult to verify, which may obscure a patient’s clinical picture and potentially lead to misdiagnosis.

Given that the translatability to telehealth score and the preceding inductive data analysis to develop the score were conducted by the same group of authors, there is a potential confirmation bias, despite attempts to remain objective throughout. For this reason, the authors would advocate for the translatability to telehealth score to be applied to different patient populations to ascertain its efficacy.

Finally, it is difficult to capture the complexities of human interactions within a scoring system, as a lot of subtleties (for example, body language) can be lost. This is a potential limitation of telehealth, as it could negatively impact the development of patient and practitioner rapport. However, given human behaviour and interactions are highly variable, it is difficult to make the scoring system more specific, which suggests that the primary benefit of the translatability to telehealth score is as a broad categorisation tool.

### Comparison with existing literature

A literature review demonstrated that telehealth can be just as effective as in-person appointments in certain contexts. Fox *et al* found that regularly scheduled telehealth appointments, with an ‘alert system’, in exacerbation-prone patients with COPD reduced the number of unscheduled GP consultations and reduced associated healthcare costs.^22^ Phillips *et al* found that there was no significant difference in the rate of related follow-ups, including hospital admissions and emergency department visits, in respiratory patients that had undergone either an initial telehealth visit or in-person visit.^15^ Totten *et al* conducted a systematic synthesis of 58 articles and found telehealth improved patient outcomes in chronic health conditions, including respiratory conditions.^23^

Davis *et al* assessed perceptions regarding telehealth in patients with cystic fibrosis and found over 70% were satisfied with their telehealth experience, but some expressed concerns regarding a lack of in-person investigations (for example, sputum sample).^24^ Clinicians’ perspectives on telehealth remain divided. Althobiani *et al* found that clinicians thought telehealth was a useful tool to monitor patients with interstitial lung disease, albeit recognising that further research pertaining to clinical outcomes was required.^25^ Phimphasone-Brady *et al* noted that telehealth introduces new barriers and exacerbates some disparities, such as negatively impacting those of lower socioeconomic backgrounds, older individuals that may be unfamiliar with technology, and patients of non-English speaking backgrounds.^26^

### Implications for research and practice

Given the rapid uptake of telehealth in recent years, new guidelines and recommendations have been developed to assist clinicians to conduct telehealth consultations.^14,27^ This study’s clinical findings could assist with the development of such resources, by quantifying which tasks are amenable to telehealth.

Some equipment is not readily available in a patient’s home and thus may not be amenable to telehealth at this point in time (for example, spirometry). However, further research and development in medical technology could allow for some of these measures to be made replicable in a home environment in the future (for example, compact spirometry devices). Despite advances in technology, however, some measures may still be difficult to translate to telehealth, such as physical examinations.

It is important to consider the impact of technology on the GP–patient relationship. In some consultations, in-person appointments facilitated the GP–patient therapeutic relationship, which was conducive to further management (for example, opportunistic smoking cessation). Further research to investigate if this translates to the context of telehealth would be beneficial.

To determine the efficacy of the translatability to telehealth score as a broad categorisation tool, it would be useful to apply the scoring system to different patient populations (for example, other health conditions and the wider respiratory population).

In conclusion, this article aimed to assess what in-person tasks were occurring in respiratory consultations with GPs, and whether these were amenable to telehealth. The translatability to telehealth score was established to quantify the extent to which certain tasks were amenable to telehealth. By understanding which tasks are amenable to telehealth and which tasks are not, clinicians would be better positioned to understand which tasks can be conducted remotely.

While telehealth appointments are beneficial, there are some instances where in-patient appointments are still required (for example, in patients of lower socioeconomic background and who have difficulty navigating technology). Thus, it is important that in-person GP appointments remain an option.

## Data

See Seguin *et al* ^17^ for details on access to study data.
